# Through the Looking Glass: The Role of Ethnicity and Affiliation in Responses to Terrorism in the Media

**DOI:** 10.3389/fpsyg.2016.01911

**Published:** 2016-12-05

**Authors:** Anat Shoshani, Michelle Slone

**Affiliations:** ^1^Baruch Ivcher School of Psychology, Interdisciplinary Center HerzliyaHerzliya, Israel; ^2^School of Psychological Sciences, Tel Aviv UniversityTel-Aviv, Israel

**Keywords:** terrorism, media, stereotypes, attitudes, anxiety, ethnicity, culture, conflict

## Abstract

This study examined whether attitudinal and emotional responses to broadcasts of images of terrorist events differ according to ethnic group (Jewish and Arab Israelis) and outgroup affiliation during an intense wave of terrorism that occurred in Israel during 2015. Participants were 118 Jewish and 110 Arab-Israelis adults randomly allocated to a terrorism or criminal violence television broadcast. State anxiety, state anger, stereotypes, and negative attitudes toward an adversary were examined prior and subsequent to the media exposure. Findings showed significant increases in anxiety, anger, stereotypes, and negative adversary perceptions in the terrorism exposure group compared to only anxiety increases in the criminal violence exposure. In the terrorism exposure group, Jewish participants showed greater increases in negative adversary perceptions of the Palestinians than Arab Israeli participants, but both groups showed similar significant increases in levels of anxiety and anger. Exposure to broadcasts of terrorism increased willingness to negotiate with the adversary among the Arab participants, but not among the Jewish participants. In the terrorism exposure group, both Jewish and Arab Israelis with high affiliation with the Palestinian cause showed less increases in stereotypes than those with low affiliation. Findings emphasize the role of ethnicity and outgroup affiliation in responses to media exposure to terrorism images.

## Introduction

Armed conflict based on ethnic and national affiliation has become the hallmark of the present era. In a world of blurred borders, exposure to the diversity of these conflicts is frequently concentrated into images flooding into personal homes via the media. By these means, individuals become caught up in hostilities, wars, and terrorism that are played out in distant locations (Shoshani and Slone, 2008b; Slone and Shoshani, 2014b). Identification with motives and consequences for the conflicted parties will differ according to many factors of demographic, ethnic, cultural, and social positioning that may mold psychological response to exposure (Fujioka, 2005; Slone and Shoshani, 2006b; Guttmann-Steinmetz et al., 2012). The present laboratory study examined changes in emotional and attitudinal responses to media exposure to terrorism across different ethnic groups. The study was conducted in Israel against the background of a recent wave of terrorism in the context of the Israeli–Palestinian conflict and examined differential responses to media coverage of terrorism images for Jewish and Arab Israeli viewers. The interesting positioning of these two ethnic groups, who were similar in their exposure to terrorism images but possibly different in the nature of their affiliations, could highlight the role of ethnicity in response to media exposure to terrorism broadcasting.

## Diversity In Psychological Reactions To Media Coverage Of War And Terrorism Images

War, hostilities, and terrorism may be projected into individuals’ lives either directly by victimization, loss, and acquaintance with victims, or vicariously via the media. In addition, the media may frequently be exploited as a tool in psychological warfare to impact viewers during war time (Golan and Shai, 2004; Slone and Shoshani, 2008c). Rapid developments in media technology have come to reflect the environment with increasing vividness and accessibility (Slone and Shoshani, 2008a; Slone et al., 2008). Geographical and physical distance between the frontline and the civilian population has narrowed, producing exposure that is penetrated overtly and covertly into viewers’ lives (Signorielli et al., 1995; Schlenger et al., 2002).

Although the media is rife with images of many forms of violence, terrorism constitutes a unique form of violence, based as it is on intergroup processes. Examination of violent acts can be approached from two major levels of analysis, violence as rooted in individual pathology and violence as based in intergroup processes (Louis and Taylor, 2002; Kruglanski and Fishman, 2006b).

Terrorist acts as deriving from individual pathology or demarcated psychiatric syndromes have received little research support (Kruglanski and Fishman, 2006b). However, there is much support for social-geopolitical or intergroup explanations (Louis and Taylor, 2002; Kruglanski and Fishman, 2006a). In this perspective, the underlying core of terrorism is a tactic of warfare used as a means to a specific end. It is the strategic use of terror and spreading of fear for the advancement of specific political objectives, usually related to forcing social change (Marsella, 2004). This social intergroup motivational foundation differentiates terrorism from other forms of violence, involving as it does political ideology, spreading of fear, and attracting attention to the group’s goals (Ganor, 2005). Other forms of violence, such as criminal violence, are theorized to be predominantly based on psychological processes of personal gratification, low self-control, pathology and deviance (Gottfredson and Hirschi, 1990; Pratt and Cullen, 2000). In addition, criminal violence crosses ethnic boundaries whereas terrorism involves clear divisions between ethnic, cultural, national, or religious groupings of perpetrators and victims. Therefore, dissemination of terrorist acts through the media would evoke different effects than other types of violence, such as criminal violence.

Research examining the implications of this infiltration of political conflict into civilian life via the media has demonstrated its negative psychological impact, showing resultant increased anxiety (Slone, 2000; Slone and Shoshani, 2014a), anger (Yukawa et al., 2001; Slone et al., 2009; Slone and Shoshani, 2010), pity and fury (Sontag, 2005; Slone and Shoshani, 2006a, 2008b), risk behaviors (Shoshani et al., 2014, 2016), and symptoms of Post Traumatic Stress Disorder, such as nightmares, anxiety, and fear (Joshi and Kaschak, 1998; Slone et al., 2013; Shoshani and Slone, 2016). Studies of the effects of extended media exposure to the events of September 11th revealed associated high levels of post traumatic symptoms and an assortment of severe negative emotions from acute psychological distress to more prolonged feelings of vulnerability and insecurity (Gil-Rivas et al., 2004; Marshall and Galea, 2004).

Many studies of reactions to media coverage of hostilities have documented a spectrum of effects from minimal to severe emotional reactions (Cho et al., 2003). However, most studies have not attempted to explain these inter-individual differences and fine distinctions in intensity of response. Thus, the study of effects of media exposure to terrorism images is superseded by addressing more complex issues of the risk factors that position target populations for more intense negative emotional effects.

## Ethnic Differences In Response To War Images In The Media

Many current conflicts involve hostilities between different cultural, national or ethnic groups and this raises the issue of possible ethnic group differences in responses to media coverage (Fujioka, 2005). Recognition of this issue provided the impetus for interest into ethnic differences, particularly after 9/11, and studies have identified several themes of differential reaction to media coverage of terrorism. Ethnic background was found to be a significant predictor of immediate reactions following the events of 9/11, with indirectly exposed African Americans more likely than Whites to express feelings of sadness and sympathetic sentiment toward the victims and less likely to endorse violent retaliation and other forms of reprisal (Chu et al., 2006). In general, following the 9/11 attacks, African Americans and Hispanics were found to report more trauma-related symptoms than Whites (Galea et al., 2002; Schlenger et al., 2002; Weissman et al., 2005). Explanations for these differences include hypothesized different cultural norms for coping with stress and ethnic variations in help-seeking behavior, leading to different rates of detection (Constantine et al., 2005).

Apart from possible psychological and sociological explanations for these ethnic group differences, the salience of ethnicity in national conflict could bring to the fore variations in reactions based on different affiliations with the parties portrayed. The mechanism of identification with parties portrayed in news coverage of a conflict can be explained in terms of social-identity theory that posits a motivational need to evaluate one’s ingroup positively and to devalue outgroups (Tajfel and Turner, 1979). When intergroup threat is heightened, there is a tendency to evaluate outgroup members at the extremes of various psychological characteristics and to implement differential selective processing and retention of information for ingroup as opposed to outgroup members (Hamilton and Trolier, 1986; Reynolds et al., 2000; Brewer, 2001).

Further developments in Social Identity Theory recognize that intergroup attitudes are strongly grounded in group memberships, social identities and social consensus, and embedded in wider ideological systems attached to social groups (Smith and Hogg, 2008). Following the ‘metacontrast principle,’ groups tend to conform to group norms and to endorse a prototypical group position (that is, minimizing intragroup differences and maximizing intergroup differences), while also construing the outgroup in stereotypic terms (Hogg et al., 1990; Hogg, 2000). Therefore, as a consequence of group affiliation and social comparison processes, group members tend to adopt attitudes, beliefs, behaviors and feelings that are consistent with their group (Haidt, 2001). There is evidence that over time groups can converge in their messages such that they produce attributes that are distinctive to the group which decreases ingroup heterogeneity (Postmes et al., 2000). In addition, studies have demonstrated that over time norm-consistent attitudes tend to dominate and group members who express non-normative attitudes often are discredited (Kashima, 2000; Marques et al., 2001).

Thus, different types of groups can have different psychological meanings and properties for their members, producing a variety of identity functions, social identifications, and intergroup perceptions and attitudes (Brown and Williams, 1984). The vast diversity of groups within which social identity functions emphasizes the need to consider the specific characteristics of the group, personal variables and the context in intergroup differentiation (Brown, 2000; Hornsey and Jetten, 2005). On the personal level, strength of ingroup identification is a powerful predictor of intergroup attitudes. On the contextual level, intergroup dispute and hostility can lead to increased cohesion within each of the competing groups and higher intergroup differentiation (Brown et al., 1999; Brown, 2000).

In studies of inter-nation and inter-ethnic attitudes, national identification has emerged as a consistent predictor of xenophobic attitudes (Lickel et al., 2000; Brown et al., 2001). The underlying nationalistic motive of war and terrorism could produce different levels of involvement and relevance for different ethnic groups. This would emphasize varying perceptions of the parties involved in the conflict and, in turn, may mediate emotional and attitudinal responses to media exposure to violent images of the conflict.

## Emotional And Attitudinal Responses To Terrorism Broadcasts

By its very nature, exposure to terrorism, both direct and indirect, is anxiety-provoking since it forces confrontation with existential threat. Terror management theory (TMT) posits that awareness of death induces a universal need to cope with inevitable mortality by defensively engaging in preserving particular sets of beliefs and values (Greenberg et al., 1990). In situations of life-threatening danger, rehabilitation of a sense of security necessitates defensive psychological response in the form of increased upholding of cultural worldviews, frequently manifested as devaluation of the threatening party and attribution of negative stereotypes to it (Greenberg et al., 1986; Pyszczynski et al., 2003).

Although stereotypes were traditionally considered to be stable and permanent (Lee et al., 1995), more recent research has shown their greater dynamic and fluid nature and inter-group sensitivity than previously believed (Madon et al., 2001). The media is an integral part of the dynamic social process that generates and constructs group assessments; often those of a dominant group over subordinate social groups and, therefore, could assume a central role in forging attitudes and stereotypes, especially during conflict periods (Mastro, 2003; Fujioka, 2005). An example for this was shown after the September 11 terrorist attacks when U.S. Presidential Address broadcasts involved increased stereotypical depictions of Muslims and negative images of Arabs (Merskin, 2004). In addition, qualitative analyses of several main television channels after the terrorist attacks revealed a dominance of Islamophobic, ethnocentric, and evangelical worldviews toward Arabs (Sheridan, 2006). In this way, adversary perception and stereotypes are particularly sensitive to terrorist events. Moreover, since the derogatory attitudes represent negative expectations about outgroup members, negative stereotypes often appear in conjunction with negative emotions toward the outgroup, such as anger, anxiety, and fear, and these exacerbate negative outgroup attitudes (Stephan and Stephan, 1996).

Interdisciplinary research on intergroup conflict emphasizes that trust in the adversary, empathy, willingness to seek reconciliation and negotiate, and perceptions of the threat posed by the adversary are key determinants in conflict resolution (Nadler and Liviatan, 2006; Slone and Shoshani, 2008c). Positive expressions of empathy toward an adversary will have positive effects on reconciliation in the presence of a basic level of trust in the outgroup (Nadler and Liviatan, 2006) whereas distrust and lack of empathy both propel groups into conflict (Kappmeier, 2016) and form a formidable obstacle to reconciliation between two conflict groups (Kelman, 2005). In addition, major events related to conflict such as terrorist attacks, wars, and confrontations frequently provide negative information about the adversary which leads to greater perceptions of threat, radicalization of attitudes and less willingness for reconciliation and peace negotiations (Bar-Tal et al., 2006).

However, there is still debate about the psychological effects of terrorism in a multicultural society that questions whether exposure to terrorism will lead to the same intolerance and derogation toward an adversary among members of different ethnic groups in the society. The Integrated Threat Theory of Prejudice (Stephan and Stephan, 2000) posits that there are individual differences in defensive reactions to threats in an intergroup context that are affected by the level of commitment and identification with the ingroup. Highly committed members of the ingroup exhibit more defensive responses such as prejudiced attitudes, negative stereotypes, hostility, contact avoidance, and negative expectancy toward outgroup members than less committed ingroup members whose reactions to the threats are more passive (Ellemers et al., 2002).

Although this theory deals primarily with individual differences in beliefs and values among same group members, it has high relevance for multi-cultural contexts. Multi-cultural societies are generally composed of several ethnic groups with diverse values, beliefs and levels of commitment to the majority group (Hale, 2004). This complexity increases when some of the ethnic groups are implicated in the political conflicts of the society that could contradict their sub-culture beliefs and values. The Israeli context is an example of such complexity and, therefore, offers an opportune context for the empirical study of the role of ethnicity and affiliation in determining emotional and attitudinal effects of media exposure to terrorism. Israel is a multi-cultural country, which has been exposed to a prolonged period of conflict and war within the complex constellation of the Israeli-Palestinian conflict. This focuses attention on responses in terms of the two central religious-ethnic groupings in the country – the Jewish majority and the Arab minority. The recent wave of terrorism in Israel represented a unique context for examining these issues since both Jewish and Arab Israelis were equally exposed to the risk for terrorist attacks but may have different affiliations with the Palestinian cause that lies at the base of the general conflict.

## Ethnicity And The Israeli–Palestinian Conflict

The two main constituents of the Israeli population are the Jewish majority and the Arab minority. Within Israel, the Israeli Arabs are the largest minority group, comprising 20.7% of the total population (Central Bureau of Statistics, 2015). During the War of Independence, much of the Arab population inhabiting former Palestine fled and settled in refugee camps along neighboring Arab countries, eventually constituting the Palestinian nation (Ghanem, 2001). Those who decided not to flee accepted citizenship in the Israeli state and were granted formal civic and legal equality.

The very particular relations between the two ethnic groups, Jewish and Arab Israelis, occur within the context of the protracted Israeli–Palestinian conflict. Against the background of failed peace accords, breakdown of negotiations, several military operations, and mutual hostilities, a recent increase in the violence occurred in the Israeli–Palestinian conflict starting early September 2015 and became known locally as the “Wave of Terror.” Some have attributed the increased violence to a social-media campaign inciting terrorism that may have influenced the Palestinian attackers (Israel Ministry of Foreign Affairs, 2016) or ongoing frustration over the failure of peace talks to end the decades-long occupation and the suppression of human rights. The attacks have generally been carried out by young lone attackers (Wedeman, 2015).

A summary of the key statistics during the Terror Wave reports that between September 2015 and February 2016, 30 Israelis were killed and 301 wounded with 27 sustaining serious injury. There were 91 rock-throwing incidents, 83 stabbing incidents, 22 vehicle ramming attacks, and 15 shootings. Palestinian media has reported that 167 Palestinians have been killed in the current round of violence, including 34 minors. Israeli security forces have arrested 3500 Palestinians and 1420 Palestinians have been injured (Israel Magen David Adom Ambulance Service, 2016).

Within this constellation, many Arab Israeli citizens may find themselves in a torn position of allegiance both to their Israeli citizenship and to their historical, familial, and cultural Palestinian affiliation. At the base of their status as Israeli citizens, Arab Israelis could have an overlapping mix of different identities including Israeli, Arab, Palestinian, and religious affiliation (Halabi and Sonnenschein, 2004; Suleiman, 2004). The complex geo-political context of the Middle East often produces a clash between these four identities. On the one hand, there could be a tendency to affiliate with the Palestinian cause as a legitimate struggle for independence on the basis of shared family kinship, culture and religion. On the other hand, Arab Israeli citizenship entails merging into the structures of the country and striving for equal civil rights within the Jewish Israeli state. In addition, both Jewish and Arab Israeli citizens are threatened and endangered by terrorist attacks inflicted in the country. Given the particular political context and given the kinship, historical, and politico-national relations between the Israeli Arab population and the Palestinians, it could be assumed that Jewish and Arab Israeli ethnic groups may hold different levels of affiliation with the Palestinian cause and may show different emotional and attitudinal responses to media exposure to terrorism images in this context.

## The Present Study

The present research provided an opportunity for refined understanding of the mechanism of different responses to media coverage of terrorism and the relevance of ethnicity and affiliation in this relation. This was a laboratory study that examined these two domains that have traditionally been allocated to field studies. In order to isolate empirically responses to media coverage of terrorism, the study design comprised two news coverage conditions, an experimental group exposed to terrorism images and a control group exposed to coverage of violent criminal acts in civilian contexts. The variable of affiliation with the Palestinian cause was measured before exposure. In a repeated measures design, the variables of state anxiety, state anger, stereotype attribution, and adversary perceptions were administered in a pre-test prior to media exposure and in a post-test subsequent to exposure.

In view of the complex constellation of ethnicity and affiliation with the Palestinian cause and perceptions of media coverage of terrorism images, the present study tested two hypotheses.

The first hypothesis expected a significant interaction of time × exposure group × ethnicity on the emotional and attitudinal responses to the media coverage of terrorism. Based on the unique aim of terrorism to instill fear and insecurity and to change attitudes among the public, we predicted that in the terrorism exposure group, both Jewish and Arab viewers would show an increase in negative emotions and attitudes toward the adversary. However, we assumed that the Jewish Israeli viewers would report a greater pre- to post-exposure increase in state anxiety, state anger, stereotypes, and negative adversary perceptions, as opposed to lower levels of emotional and attitudinal changes among the Arab Israeli viewers. This hypothesis was based on the assumption that the multi-component Arab Israeli identity may moderate the increase in negative emotions and attitudes toward the adversary.

Since criminal violence does not contain a clear inter-group component, we predicted a different pattern of time × ethnicity interaction. In the criminal violence group, we predicted lower pre- to post-exposure changes in state anxiety and anger among viewers, and no significant attitudinal changes toward the adversary, compared to the terrorism exposure group, with no significant differences between Arab and Jewish Israelis.

The second hypothesis predicted that affiliation with the Palestinian cause will moderate attitudinal responses in the terrorism exposure group but not in the criminal violence group, such that participants with high affiliation will show less negative attitudinal increases than those with low affiliation. This hypothesis was based on the theoretical assumptions of Integrated Threat Theory (Stephan and Stephan, 2000) that level of identification with the ingroup values and agendas will affect defensive reactions to inter-group threats.

## Materials and Methods

### Participants

Participants were 228 young adult Jewish and Arab Muslim students from an academic institution in Israel, aged 18–28 (mean age = 21.74, *SD* = 2.15) and approximately evenly divided by gender, who were recruited through the campus website for research participation credit on a voluntary basis. Except for controlling for gender divisions, participants were randomly allocated into a terrorism exposure (*n* = 114) or criminal violence condition (*n* = 114) until equal numbers from each ethnic group were reached in each exposure group (Jewish Israelis = 59; Arab Israelis = 55 in each group). The study population consisted of a majority from middle socio-economic status (68%) and others from low SES (17%) and high SES (15%) groups. 58% defined themselves as secular, 22.6% as traditional, and 19.4% as religious. 96.7% were unmarried. Except for ethnic group and religion, comparisons of the two ethnic group samples, Jewish and Arab, showed no significant differences in other demographic characteristics. There were no significant differences in age, gender, socio-economic status, and marital status between participants in the terrorism exposure and criminal violence groups.

### Instruments and Procedures

#### Exposure Manipulation

The exposure manipulation consisted of two 7-min movie clips, one for the terrorism and the other for the criminal violence exposure. A team consisting of the experimenters and a computer expert who was trained and proficient in media materials and communications. prepared both clips for the study. Clips consisted of items extracted from accumulated recorded material that had been broadcast in news programs since September 2015. In the terrorism exposure condition, perpetrators were Palestinians and victims were both Jewish and Arab Israelis. In the criminal violence condition, both perpetrators and victims were Jewish and Arab Israelis. The terrorism and criminal violence clips were matched on parameters of length, the same male and female reporters, and television channels on which news items had been aired. For the terrorism clip, the material showed damage incurred by terrorist attacks in Israel that involved stabbings, shootings, and vehicular ramming attacks. For the criminal violence clip, the material showed recent news items containing images of criminal events and casualties in public places in Israel including gang assassinations, robbery and stabbings in clubs. The clips were compiled so that items flowed smoothly across the sections and the final products were equivalent in terms of length of discrete items, proportion of editorial presentation to graphic visual material, and proportion of background music to verbal report.

#### Anxiety

Anxiety was measured with the state anxiety section of the STAI (State-Trait Anxiety Inventory; Spielberger et al., 1970) comprised of 20 descriptions of anxiety-related emotional states that participants rate for the extent items reflect their current state on a 4-point Likert scale from 1 (not at all) to 4 (very much). The inventory is scored as the sum of ratings attributed to each item. Scores range from 20 to 80, with higher scores reflecting higher levels of state anxiety. In this study, psychometric properties were acceptable with internal consistency scores for the pre-test α = 0.94 and for the post-test α = 0.96.

#### Anger

Anger was measured with the state anger section of the STAXI (State-Trait Anger Expression Inventory; Spielberger, 1988), comprised of 10 items that assess the intensity of anger as an emotional state at a particular time. Participants rate items on a Likert-type scale from 0 = not at all to 4 = very much. The inventory is scored as the sum of ratings attributed to each item. Final scores range from 10 to 40, with higher scores reflecting higher levels of state anger. In the current study, Cronbach’s alpha coefficients were 0.92 for the pre-test and 0.91 for the post-test.

#### Conflict Adversary Perception

The Conflict Adversary Perception Questionnaire was originally developed (Bar, 1999, unpublished) and has been used (Shoshani and Slone, 2008a) to examine Jewish Israelis’ perceptions of the Palestinian inhabitants of the Occupied Territories in the context of the Israeli–Palestinian conflict. In order to capture differences in perceptions across the two participant ethnic groups, some items were reworded to avoid allusion to either of the parties in the conflict as enemies. The questionnaire contains 38 items relating to trust and empathy toward the adversary, willingness to negotiate, and perceptions of Palestinian hostility toward Israel. Respondents rate items for agreement on a Likert scale from 1 = do not agree at all to 7 = agree completely, and the total score is the sum of ratings. The subscale of trust includes seven items and its total score ranges from 7 to 49. The subscale of empathy toward the adversary includes 11 items summed to a total score that ranges from 11 to 77. The subscale of willingness to negotiate with the adversary includes 15 items and the summed total score ranges from 15 to 95. The subscale of perceptions of Palestinian hostility consists of five items and the total hostility score ranges from 5 to 35. The internal reliability coefficient in the present study was α = 0.87 for the pre-test and α = 0.91 for the post-test.

#### Stereotype Attribution

In order to measure attribution of stereotypes, we used a Differential Semantic Technique (Suci and Osgood, 1957) that has been used in previous studies to measure ethnic stereotypes (Slone et al., 2000). In the present study, the inventory presented descriptions of a target population referred to as the Palestinians in the Occupied Territories. The Semantic Differential Inventory used 24 items of opposing sets of characteristics, such as extremist – moderate, moral - immoral, set along a 7-point scale that respondents rated for agreement regarding the target population. The inventory is scored as the sum of agreement ratings across all sets of characteristics. Scores range from 24 to 168, with higher scores reflecting higher levels of stereotypes. The inventory yielded good psychometric properties with internal consistency coefficients of α = 0.88 for the pre-test and α = 0.92 for the post-test.

#### Affiliation with the Palestinian Cause

This questionnaire was originally developed and has been used to examine attitudes toward peace in the context of the Israeli–Palestinian conflict (Nachtwey and Tessler, 2002). We selected six items from the original 13 items to assess affiliation with the Palestinian cause. Example items include “Palestinians deserve their own homeland – an independent state,” “Israel should agree to the establishment of an independent Palestinian state,” and “Palestinian independence is the natural right of the Palestinian people.” Respondents rate items on a 4-point scale ranging from 1 = strongly disagree to 4 = strongly agree. The sum of the six items was calculated and ranged from 6 to 24, with higher scores indicating more affiliation with the Palestinian cause. In the current study, Cronbach’s alpha coefficient was 0.86.

### Procedure

The experiment, authorized by the Tel Aviv University Ethics Committee, was conducted in a quiet pleasant laboratory from November 2015 to January 2016. Participants gave written consent for their participation. Refusal rate was zero. The computerized procedure on Qualtrics was completed individually. At the beginning of the experiment, participants completed a battery of questionnaires that included the personal details questionnaire, the Affiliation with the Palestinian Cause Questionnaire (Nachtwey and Tessler, 2002), State-Trait Anxiety Index (Spielberger et al., 1970), State-Trait Anger Expression Index (Spielberger, 1988), Adversary Perception Questionnaire (Bar, 1999, unpublished), and Stereotype Attribution Inventory (Slone et al., 2000). After completion of the pre-test battery, equal numbers of Jewish and Arab Israeli participants were automatically randomly assigned into one of the two exposure conditions, the experimental terrorism or control criminal violence exposure. Immediately after viewing the assigned clip on the computer, participants completed the post-test battery that included the same pre-test measures, except for the personal details and the Affiliation with the Palestinian Cause Questionnaires. In both pre- and post-test batteries, questionnaires were presented in a randomly counter-balanced order to limit response bias. At the end of the experiment, experimenters debriefed the participant. Care was taken to avoid termination of the experiment with participants in distress.

### Data Analyses

To test the first hypothesis, a mixed ANOVA was performed. The two between-subject factors were ethnic group (Jewish/Arab) and type of exposure (terrorism exposure/criminal violence). The within-subject factor was time (time 1: pre-exposure, time 2: post-exposure) and the dependent variables were state anxiety, state anger, trust in the adversary, empathy toward the adversary, willingness to negotiate with the adversary, perception of adversary hostility, and stereotype attribution of the adversary.

For the second hypothesis, the moderation effects of affiliation to the Palestinian cause were examined by the PROCESS macro for SPSS developed by Hayes (2013). The mean ± 1 standard deviation of the moderator (i.e., affiliation to the Palestinian cause) was used to examine its conditional effects (Hayes, 2013).

The SPSS Missing Value Analysis package was used to estimate the pattern of missing data and impute missing values with appropriate procedures. Missing data was less than 2% of the total data and a multiple imputation method was applied to replace the missing values. Possible baseline differences between ethnic groups were tested using *t*-tests for independent samples. Statistically significant effects were corroborated with effect size measures, specifically with $\eta_{p}^{2}$ and Cohen’s *d*. According to recommendations by Cohen (1988), Cohen’s *d* effect sizes of 0.20 were interpreted as small, 0.50 as moderate, and 0.80 as large.

## Results

### Preliminary Data Analyses and Descriptive Statistics

Preliminary data analyses showed that all the study variables were normally distributed with no unusual kurtosis or skewness. **Table 1** provides descriptive statistics by type of exposure and ethnic group for the emotional and attitudinal variables prior and subsequent to the exposure manipulation. Possible baseline differences between ethnic groups were tested using *t*-tests for independent samples. There were no significant baseline differences between Arab and Jewish Israelis for state anxiety, state anger, and trust in the adversary. However, there were statistically significant baseline differences in stereotype attribution, *t*(226) = 6.11, *p* < 0.001, empathy toward the adversary, *t*(226) = 3.58, *p* < 0.001, willingness to negotiate with the adversary, *t*(226) = 2.45, *p* = 0.02, and perception of adversary hostility, *t*(226) = 3.60, *p* < 0.001, with lower levels of stereotypes and perception of adversary hostility and higher levels of empathy and willingness to negotiate with the Palestinians among Arab Israelis compared to Jewish Israelis. In addition, findings showed significant differences in affiliation with the Palestinian cause between Jewish and Arab Israelis such that Arab Israelis reported significantly greater affiliation with the Palestinian cause (*M* = 14.13, *SD* = 4.34) than Jewish Israelis (*M* = 11.95, *SD* = 3.79), *t*(226) = 4.06, *p* < 0.001.

**Table 1:** Means and standard deviations for pre- and post-test levels of the dependent variables for each type of media exposure and ethnic affiliation.

Dependent variables	Ethnic affiliation	Terrorism exposure (*n = 114*)					Criminal violence exposure (*n = 114*)				
		Time 1		Time 2			Time 1		Time 2		
		*M*	*SD*	*M*	*SD*	*D*	*M*	*SD*	*M*	*SD*	*D*
State anxiety	Jewish	38.29	7.65	43.94	5.85	0.83	39.40	9.12	41.88	6.27	0.32
	Arab	40.37	8.20	46.11	6.69	0.77	41.15	7.89	43.47	5.48	0.34
State anger	Jewish	25.94	8.55	31.93	6.06	0.81	24.96	7.86	26.61	5.54	0.24
	Arab	26.10	8.15	31.60	5.74	0.78	25.80	9.10	27.46	7.01	0.20
Stereotype attribution	Jewish	91.24	8.47	95.82	7.15	0.58	93.81	7.41	92.85	6.88	0.13
	Arab	87.39	6.51	90.58	7.25	0.46	85.92	6.34	86.60	6.90	0.10
Trust in the adversary	Jewish	26.25	5.28	21.76	4.94	0.88	25.75	5.19	26.41	4.82	0.13
	Arab	24.20	5.33	21.30	5.58	0.53	25.37	4.62	25.63	3.69	0.06
Empathy toward the adversary	Jewish	53.62	6.64	49.80	5.75	0.62	52.15	5.90	51.67	7.01	0.07
	Arab	55.25	6.76	52.27	5.35	0.49	56.80	7.15	57.41	6.90	0.09
Willingness to negotiate with the adversary	Jewish	70.41	8.75	71.67	7.80	0.15	72.15	7.93	73.08	7.62	0.12
	Arab	74.20	7.90	79.80	7.15	0.74	73.67	8.10	72.90	8.40	0.09
Perception of adversary hostility	Jewish	23.14	4.10	25.65	4.48	0.58	21.80	4.67	22.10	4.53	0.07
	Arab	19.87	5.01	23.10	5.36	0.62	20.60	4.95	20.35	5.10	0.05
Affiliation with the Palestinian cause	Jewish	11.65	3.61				12.24	3.97			
	Arab	14.36	4.52				13.90	4.15			

*Time 1 and Time 2 refer to measurement before or after media exposure; n = 59 for Jewish Israelis, n = 55 for Arab Israelis in each exposure group.*

### Interaction Effects of Time, Type of Exposure and Ethnicity

The first hypothesis that assumed a significant interaction effect of time, ethnicity (Jewish/Arab Israelis) and type of media exposure (terrorism exposure/criminal violence) on emotional and attitudinal changes was tested by a mixed ANOVA analysis.

The time × type of exposure effects for all the emotional and attitudinal measures in the study were significant, indicating a significantly different level of change between exposure groups (terrorism and criminal violence) in state anxiety, *F*(1,224) = 11.48, *p* = 0.001, $\eta_{p}^{2}$ = 0.05, state anger, *F*(1,224) = 18.66, *p* < 0.001, $\eta_{p}^{2}$ = 0.08, stereotype attribution, *F*(1,224) = 13.15, *p* < 0.001, $\eta_{p}^{2}$ = 0.06, trust in the adversary, *F*(1,224) = 16.18, *p* < 0.001, $\eta_{p}^{2}$ = 0.07, empathy toward the adversary, *F*(1,224) = 4.02, *p* = 0.04, $\eta_{p}^{2}$ = 0.02, willingness to negotiate with the adversary, *F*(1,224) = 19.57, *p* < 0.001, $\eta_{p}^{2}$ = 0.08, and perception of adversary hostility, *F*(1,224) = 19.83, *p* < 0.001, $\eta_{p}^{2}$ = 0.08. More specifically, in both exposure groups there were significant increases in state anxiety, but with significantly greater increase in the terrorism exposure group [mean change = 5.70, *t*(113) = 6.02, *p* < 0.001, *d *= 0.80], compared to the criminal violence group, [mean change = 2.4, *t*(113) = 2.48, *p* = 0.01, *d *= 0.33]. In addition, in the terrorism exposure group, there were significant increases in state anger [mean change = 5.80, *t*(113) = 6.01, *p* < 0.001, *d *= 0.81]; stereotype attribution [mean change = 3.89, *t*(113) = 3.99, *p* < 0.001, *d *= 0.53]; perception of adversary hostility [mean change = 2.87, *t*(113) = 4.57, *p* < 0.001, *d *= 0.61); and willingness to negotiate with the adversary (mean change = 3.44, *t*(113) = 3.80, *p* = 0.001, *d *= 0.44], and decreases in trust [mean change = -3.69, *t*(113) = 5.27, *p* < 0.001, *d *= 0.70], and empathy [mean change = -3.41, *t*(113) = 4.18, *p* < 0.001, *d *= 0.56]. Effect sizes for the significant changes were in the moderate to large range (Cohen, 1988). In the criminal violence group, there were no significant changes in state anger and attitudes toward the adversary.

The results yielded also a significant three-way interaction of time × exposure group × ethnicity on stereotype attribution, *F*(1,224) = 5.06, *p* = 0.03, $\eta_{p}^{2}$ = 0.02, trust in the adversary*, F*(1,224) = 4.33, *p* = 0.04, $\eta_{p}^{2}$ = 0.02, empathy toward the adversary, *F*(1,224) = 4.15, *p* = 0.03, $\eta_{p}^{2}$ = 0.02, and willingness to negotiate with the adversary, *F*(1,224) = 12.74, *p* < 0.001, $\eta_{p}^{2}$ = 0.05. The terrorism exposure was associated with increased stereotype attribution and reduced trust and empathy toward the adversary in both Jewish and Arab groups. However, these changes were more pronounced and significantly greater among the Jewish participants than among the Arab participants. A different pattern emerged for willingness to negotiate with the adversary. The terrorism exposure was associated with increased willingness to negotiate with the adversary among the Arab participants, *t*(54) = 3.90, *p* < 0.001, *d* = 0.74, but no significant change emerged among the Jewish participants, *t*(58) = 0.82, *p* = 0.41, *d* = 0.15. Finally, in the terrorism exposure, there were increases in state anxiety, state anger, and perception of adversary hostility, with no significant differences between Arab and Jewish participants. In the criminal violence group, in accordance with the hypothesis, there were no significant differences between Jewish and Arab participants in pre- to post-exposure emotional and attitudinal changes.

### The Moderation Role of Affiliation with the Palestinian Cause

In order to test for the moderation effect of affiliation with the Palestinian cause on the effect of type of media exposure (terrorism exposure/criminal violence) on attitudinal and emotional responses, we performed moderation analyses by PROCESS (Hayes, 2013). Pre- to post-exposure change scores were employed as dependent variables. Results yielded a significant interaction between affiliation with the Palestinian cause and type of media exposure only for the stereotype attribution measure (*b* = -0.31, *SE* = 0.05, *t* = -6.32, *p* < 0.001). To understand the nature of this interaction, we examined the stereotype attribution change as a function of type of exposure and affiliation with the Palestinian cause at 1 standard deviation above and below the mean (Cohen and Cohen, 1983). The effect of type of media exposure on stereotype attribution was significantly higher among participants who were 1 standard deviation below the mean of affiliation with the Palestinian cause (*b* = 0.69, *SE* = 0.06, *t* = 11.95, *p* < 0.001) than among participants who were 1 standard deviation above the mean of affiliation with the Palestinian cause (*b* = 0.27, *SE* = 0.07, *t* = 3.90, *p* < 0.001). Affiliation with the Palestinian cause did not moderate the other study variables.

## Discussion

The construction of a laboratory study enabled drawing apart the role of ethnicity and affiliation in positioning individuals differently for emotional and attitudinal responses to the violent scenes of terrorism that penetrate into personal lives via the media. The first hypothesis predicting a significant interaction between exposure group and ethnic group on pre- to post-exposure changes in the emotional and attitudinal responses was partially confirmed.

Regarding the difference between the two exposure conditions, as expected, participants in the terrorism exposure group exhibited significantly higher increases in state anxiety, state anger, stereotypes, and negative adversary perception than those in the criminal violence group. These differences could be explained by the images in the terrorist clip that could easily arouse psychological proximity for the Israeli viewer. Psychological proximity is defined in terms of similarity between viewers and depicted characters or events by means of identification (Shoshani and Slone, 2008b). Portrayal of routine situations that became terrorist sites and possible demographic resemblance between victims and some viewers may have intensified psychological proximity mechanisms. The visual material reflecting dangerous real life situations that are contained in terrorism broadcasts could activate identification mechanisms and subjective emotional experience (Reeves and Nass, 1996). These could heighten the mortality salience of the images, making the threat more concrete and personally relevant. The security threat in Israel is highly salient and omnipresent in all aspects of life. On the other hand, the criminal violence images could be perceived as more distant and less personally relevant because criminal violence is not a pervasive issue in Israel and far fewer individuals are involved in this form of violence. In addition, this finding indicates the intensity of fear evoked by the terrorist images in comparison to the criminal violence exposure. This finding reflects the motivation of terrorism aiming to arouse anxiety, fear and reduced sense of security as a tactic of warfare.

The prediction regarding the effects of ethnicity on pre- to post-exposure changes in emotional and attitudinal responses to the terrorism as opposed to the criminal violence exposures was partially confirmed. In the terrorism exposure group, results revealed similar increases in levels of anxiety, anger, and perception of adversary hostility for Arab and Jewish participants. However, for the attitudinal variables, a steep increase was found in stereotypes and a steep decrease in trust and empathy toward the adversary in both groups, but larger changes occurred among Jewish than Arab Israelis.

The intensity of emotional reactions of both ethnic groups signifies reactions to viewing the harsh realities of the intractable conflict. Both ethnic groups showed similar emotional responses to the threat of being potential targets of unexpected, unpredictable attacks in routine daily sites. A sudden exposure to these images could trigger a reminder of the insecurity in the country.

However, the different attitudinal changes in the two ethnic groups reflect the salience of ethnicity in the complex conflict. Arab Israelis showed less increases in stereotype attributions and negative adversary perceptions. Given the complicated geo-political environment in Israel and the sensitivity of intergroup relations, Jewish and Arab Israelis could show very different attitudes regarding the Israeli–Palestinian conflict. The minority status of Arab Israelis and their multi-component identities (Ellemers et al., 2002) could lead to different ingroup-outgroup categorizations than their Jewish Israeli counterparts, such that perceptions of Palestinians in the Occupied Territories are not clearly differentiated as an outgroup. Mutual cultural, religious, kinship and historical bonds could blur boundaries and increase involvement and affiliation with the Palestinian cause.

For the Arab Israelis, exposure to terrorism images did not necessarily lead to the same reductionism of features regarding the Palestinian people that occurred for the Jewish Israeli participants. The Arab Israeli participants did not resort to the same level of extreme stereotyping as the Jewish participants exhibited. A striking difference that emerged between the two ethnic groups related to willingness to negotiate with the adversary. In the terrorism exposure group, Arab participants reported greater willingness to negotiate with the adversary than Jewish participants who did not show significant changes in willingness for reconciliation. Jewish participants showed greater extremism in attitudes toward the adversary and less flexibility for attitude change regarding negotiations for reconciliation. Arab participants showed greater attitudinal flexibility and increased willingness to engage in negotiations and to seek out resolution of the conflict. These findings highlight the difficult positions in which different ethnic groups find themselves when confronted by terrorism in a conflict environment. In this case, for Jewish and Arab Israeli groups, this position includes the common fate of being potential victims of terrorist attacks while holding different attitudes toward the nature, process and options for reconciliation of the conflict.

The second hypothesis predicted that affiliation with the Palestinian cause will moderate attitudinal and emotional responses in the terrorism exposure group but not in the control group. The hypothesis was confirmed only in relation to stereotypes, such that participants with low affiliation with the Palestinian cause showed greater increases in stereotype attribution than those with high affiliation. This finding suggests the prominent role that can be played by affiliation as a process that moderates prejudice attitudes to charged media images of terrorism.

In the terrorism exposure group, affiliation with the legitimacy of the Palestinian protest did not moderate the emotional responses or perceptions of the adversary hostility. However, this affiliation did moderate informational distortion about the adversary as reflected in stereotype attribution. Many intergroup perceptions are not based on objective group information but rather are dependent on bias. Research on ingroup-outgroup categorization has shown that there is a tendency to evaluate outgroup members at the extremes of various psychological characteristics and to implement different selective processing and retention of information for ingroup as opposed to outgroup members (Tajfel and Turner, 1979; Hamilton and Trolier, 1986). In this study, in the terrorism exposure group, it seems that political attitudes functioned as a buffer against selecting only negative information and, therefore, enabled more objective reception of the information provided by the broadcasts.

The findings of the current study suggest that dichotomous ingroup-outgroup divisions become far more complicated in a multi-cultural environment in which members of the different group dissent in their attitudes, opinions, values and identities. The complexity of such a multi-cultural environment could possibly be represented as comprising circles of groups and identities that preserve their unique features in some situations and overlap with different groups and identities in other situations. This would present some explanation for the complexity of emotional and attitudinal responses of different groups in contexts of war, conflict, and terrorism.

### Limitations and Implications

Participants in this study were students thus limiting generalization of results. In the terrorism exposure condition, the perpetrators were Palestinian whereas in the control criminal violence condition, the perpetrators were both Jewish and Arab Israelis. Ideally, perpetrators in the two exposure conditions should have been equivalent with regard to ethnicity and conditions should have differed only with respect to terrorism as opposed to criminal violence. However, it was not possible to find actual material of Palestinian criminal violence against Israelis equivalent in the intensity of violence (stabbing, shooting, vehicular ramming at a bus stop) with criminal intention because this rarely, if ever, occurs in Israel. Future studies of this type should find contexts that allow for balancing ethnic salience across conditions.

In order to examine the unique effects of terrorism in the media as opposed to other forms of violence, the present study included one control condition consisting of exposure to criminal violence. An additional violence-free neutral control condition could have reflected the effects of media presentation of violence in itself and the greater intensity of terrorism above criminal violence. Future studies should consider inclusion of an additional violence-free control group in the study design.

The strength of this study is also related to its weakness. On one hand, the present research makes a unique contribution in providing a rigorous laboratory methodology that has been largely lacking in much previous research in this field. On the other hand, conditions in a laboratory study would differ from a real life situation in which terrorism images are projected in real time and impart actual information. Nevertheless, the laboratory condition did produce a change in attitudes both within and between groups, reinforcing the central role of ethnicity and affiliation in this context. A potential confound in this study is that the criminal violence control clips may have also aroused mortality salience, as in the terrorism exposure. Thus, this control condition could have led to similar attitudinal and emotional changes. However, exposure to terrorism is a very specific type of mortality salience especially in environments of protracted conflict. In Israel, the mortality salience involved in media exposure to terrorism is related to actual threat and is related overtly to a specific outgroup perceived as threatening. The specificity of the terrorism exposure in Israel would explain the different emotional and attitudinal responses to the terrorism as opposed to the criminal violence exposures.

Despite the specific political background of the intractable Israeli–Palestinian conflict, this multi-cultural context represented an opportune context for the empirical study of different effects of media exposure to terrorism and the role of ethnicity and affiliation in molding responses. Further research should extend this perimeter to other conflict contexts and to examination of these factors in different cultural compositions.

The media finds itself at the interface between portrayal of terrorism and its impact on viewers of different affiliations and creeds. Acknowledgment and sensitive analysis of different responses based on these divisions enables deeper comprehension of reactions to media exposure to terrorism and adds a multi-cultural perspective to attempts for conflict resolution.

## Author Contributions

AS and MS have substantial contributions to the conception of the study, the acquisition, analysis, and interpretation of the research data; and preparing the manuscript for publication.

## Conflict of Interest Statement

The authors declare that the research was conducted in the absence of any commercial or financial relationships that could be construed as a potential conflict of interest.
